# Imbalance in Individual Researcher's Peer Review Activities Quantified for Four British Ecological Society Journals, 2003-2010

**DOI:** 10.1371/journal.pone.0092896

**Published:** 2014-03-21

**Authors:** Owen L. Petchey, Jeremy W. Fox, Lindsay Haddon

**Affiliations:** 1 Institute for Evolutionary Biology and Environmental Studies, University of Zurich, Zurich, Switzerland; 2 Department of Biological Sciences, University of Calgary, Calgary, Alberta, Canada; 3 British Ecological Society, London, United Kingdom; Université de Montréal, Canada

## Abstract

Researchers contribute to the scientific peer review system by providing reviews, and “withdraw” from it by submitting manuscripts that are subsequently reviewed. So far as we are aware, there has been no quantification of the balance of individual's contributions and withdrawals. We compared the number of reviews provided by individual researchers (i.e., their contribution) to the number required by their submissions (i.e. their withdrawals) in a large and anonymised database provided by the British Ecological Society. The database covered the Journal of Ecology, Journal of Animal Ecology, Journal of Applied Ecology, and Functional Ecology from 2003–2010. The majority of researchers (64%) did not have balanced contributions and withdrawals. Depending on assumptions, 12% to 44% contributed more than twice as much as required; 20% to 52% contributed less than half as much as required. Balance, or lack thereof, varied little in relation to the number of years a researcher had been active (reviewing or submitting). Researchers who contributed less than required did not lack the opportunity to review. Researchers who submitted more were more likely to accept invitations to review. These finding suggest overall that peer review of the four analysed journals is not in crisis, but only due to the favourable balance of over- and under-contributing researchers. These findings are limited to the four journals analysed, and therefore cannot include researcher's other peer review activities, which if included might change the proportions reported. Relatively low effort was required to assemble, check, and analyse the data. Broader analyses of individual researcher's peer review activities would contribute to greater quality, efficiency, and fairness in the peer review system.

## Introduction

The quantity of articles published in scholarly journals increased by 200 to 300% from the early 1980s to the late 1990s [1]. Although it seems likely that also the number of available reviewers increased [2], concerns remain about increases in reviewing load [3]–[5], and the possibility of a peer review system in crisis [6]. Such concerns often suggest that the peer review system is susceptible to the tragedy of the commons [7], in which individual behaviour can worsen the situation of a group [8] (though the exact analogy is questionable, depending on if the commons is managed or unmanaged [9]). The peer review system also has been characterised as a “reciprocal altruistic system”, since the same researchers are both authors and reviewers [3]. Ideally, researchers should balance the “withdrawals” they make from the “peer review commons” as authors by contributing the appropriate number of reviews [3], [6].

There are suggestions that some of the most active publishing scientists tend to be least likely to review [8], [10], though such individuals may be quite rare [3]. We know, however, of no quantitative assessment of this phenomenon. More broadly, we know of no quantitative studies of how well individual researchers balance the number of reviews they contribute to the peer review system with the number of reviews they require of the system.

A recent survey based study found that the although individual researchers' “decline to review rate” (number of declined review requests divided number of requests) increased with the number of papers published per year, so did the number of review requests, such that number of reviews also increased [11]. However, the information reported did not address whether individuals balance their “withdrawals” and contributions to the peer review system, since it concerned only number of published papers. Nor did it address the variability among individuals in their balance of withdrawals and contributions. Finally, there was considerable variation in the reported relationship between number of reviews provided and number of papers published.

Here we report a quantitative comparison of individual researchers' balance of reviewing and submission activities for data from four journals of the British Ecological Society over the period 2003–2010.

## Methods

### Ethics Statement

The appropriate body of the University of Zurich does not consider this study to require their ethical approval.

### Data availability

The British Ecological Society, given the anonymised nature of the data, has given permission for the data to be openly accessible (data available from the Dryad Digital Repository: http://doi.org/10.5061/dryad.36r69).

We used data provided by the British Ecological Society covering the years 2003–2010 for the Journal of Ecology, Journal of Animal Ecology, Journal of Applied Ecology, and Functional Ecology. One database contained the 13068 reviewed original submissions received during the 8 years, made by 7566 submitting authors. Each submission record included the year of submission, the journal submitted to, the anonymised identity of the submitting author, the total number of authors, the number of reviewers invited, the number of reviews received, and the editorial decision. We had no information about the identity of coauthors, anonymised or otherwise. The other database contained the 14294 researchers invited to review at least once, across the four journals. Each reviewer record contained the anonymous identity of the reviewer, the year, the reviewer's country, the number of invitations to review, the number of completed reviews, and the inviting journal. Since we were interested in the balance between the submission and reviewing activities of individual researchers, we restricted our analyses to the 4055 individual researchers who acted as both submitting author and invited reviewer. (3511 submitting authors were never invited to review. 10239 invited reviewers were never submitting authors.)

From these data we calculated for each individual researcher the number of reviews they provided (simply by summing the number of reviews provided during the eight years) and the number of reviews that were required by their submission(s). The latter calculation was somewhat complex due to the presence of coauthors. We first calculated the average number of reviews per submission (i.e., total number of reviews performed divided by the total number of manuscripts submitted, in the entire dataset). The result was 2.1 reviews per submission (77% of submissions received two reviews). We then divided the result by the number of authors of each submission, and summed this across all the submissions of a researcher. The calculation assumedthat required reviews are distributed evenly among all coauthors [12]. Coauthors rarely contribute equally to a paper [13], [14], so we also calculated number of reviews required under the assumption that the submitting author is responsible for all reviews.

Since we did not have access to the anonymised identity of coauthors, we could not assign to researchers the “withdrawals” caused by coauthorship. Therefore, the analyses presented likely underestimate the number of reviews required of the system by individual researchers. That is, our results provide only a lower bound on the proportion of researchers whose contributions to the peer review system fail to match their withdrawals, and an upper bound on the proportion of researchers whose contributions to the peer review system more than match their withdrawals.

## Results

**Figure 1:**
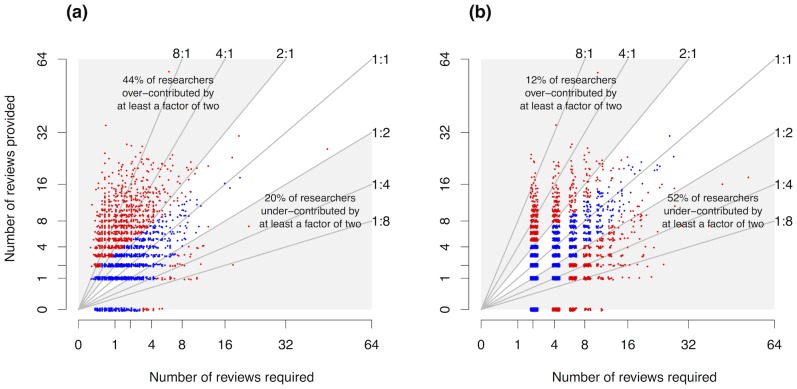
Individual researcher's peer review balance. Perfect balance would be if researchers reside on the 1∶1 line, since then they provide the same number of reviews as their submissions require. Red data points indicate individuals whose over- or under-contribution is unlikely to have resulted from chance (p>0.05, binomial test). Blue data points indicate p>0.05, though note that tests for individuals close to the origin have low statistical power. Panel (a) corresponds with the assumption that reviews are distributed equally among co-authors, (b) with the assumption that the submitting author is responsible for all reviews. Inset: dynamics of proportion of researchers over-contributing by more than double (upper line) or under-contributing by less than half (lower line). Axes of the main plot are square root scaled to better illustrate variation close to the origin. Y-values in both panels, and x-values in panel b are slightly jittered to assist visualization of otherwise overlaying points.

There was considerable variation in the relationship between the number reviews provided by researchers, and the number required by their submissions (figure 1). Assuming that required reviews are distributed evenly among coauthors, a total of 1796 (44%) researchers provided more than twice as many reviews as required by their submissions. A total of 814 (20%) researchers provided less than half as many reviews as required by their submissions. These proportions were quite invariant to variation in the year in which researchers first submitted an article (figure 1 inset). The observed variation is much greater than one would expect by chance (i.e., if researchers knew the proportion of review requests they should accept, and accepted requests with this probability) (figure 1).

If we instead assume that the submitting author is responsible for all required reviews, the proportions change considerably, to 12% providing twice as many reviews as required, and 52% providing half as many as required. Note that the sum of these two proportions is unaffected by the assumption (64% in both cases).

If we return to the assumption that required reviews are evenly distributed among authors, of the 814 reviewers who provided fewer than half as many reviews as required to balance their submissions, 668 did not provide all the reviews they were invited to. This latter group was, in total, responsible for a shortfall of 979 reviews, from a total number of reviews required of 6807.

**Figure 2:**
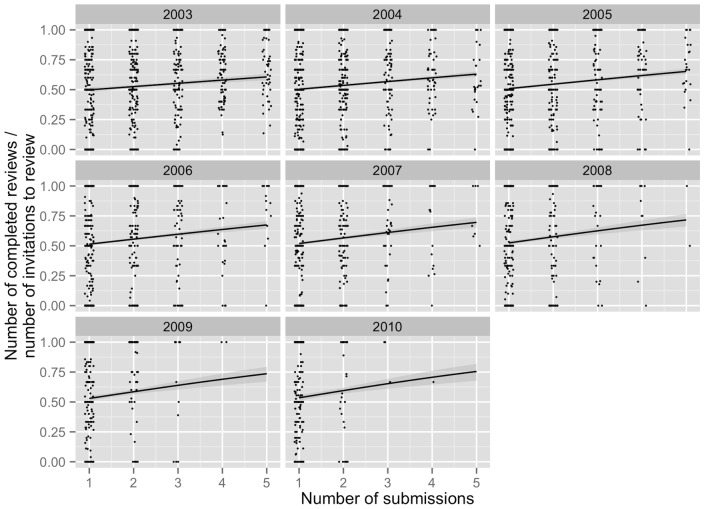
Individual researchers review completion rate, with separate graphs corresponding to the year the researchers entered the database. Lines are predictions from a quasibinomial response variable model with number of submissions, year of first submission, and their interaction, as continuous explanatory variables. The data analysed was limited to individual researchers with less than six submission, in order to avoid comparing across very different ranges of number of submissions. The model was part of an exploratory analysis, so use of p-values is inappropriate. X-axis values are jittered slightly

Researchers who submitted more papers had higher review completion rates (i.e., the number of reviews completed divided by the number of review invitations received) (figure 2). This positive relationship was stronger for researchers who had entered the database relatively recently (e.g., researchers submitting their first manuscript in years 2008–2010, compared to those who submitted their first article in years 2003–2005). This implies that these more recently-added researchers (perhaps students and postdocs) respond more sensitively to greater numbers of requests to review, by accepting a greater proportion of those requests.

## Discussion

That so many researchers provided more reviews than the number required to balance their submissions is encouraging for the peer review system. That somewhere between 20% and 52% of researchers substantially under-contribute also is encouraging. Also encouraging is the tendency for more frequent submitters to also complete a greater proportion of invited reviews.

There was considerable variation among individuals, with some providing many more, and some many fewer reviews than required by their submissions. There are numerous explanations for individuals over- or under-contributing. Quantitative analysis of what drives the behaviour of individual researchers is beyond the scope of our study. With the limited data available to us, we cannot distinguish the relative importance of alternative explanations for inter-individual variation, although we can rule out chance variation. We can also rule out lack of opportunity to review as an explanation for under-contributions, since a large majority of under-contributors declined at least one invitation to review. Applying theories of the evolution and maintenance of cooperation [15], [16] to the peer review system is an intriguing possibility, and below we identify some testable hypotheses.

Over-contributors may be motivated by a sense of professional obligation, by the incentives that some journals offer (e.g., the receipt of free access and public recognition of one's contribution offered by BES journals), by access to papers before publication, and by the opportunity to learn from the comments of the editor and other reviewers. The propensity for researchers who submit more to accept a higher proportion of reviews suggests that, at least on average, researchers believe that submitting a lot requires reviewing a lot (figure 2). The greater strength of this relationship for more recent researchers further suggests that the younger researchers may feel the obligation to balance their submissions and reviews more strongly than the older ones. Alternatively, it could be that these individuals (younger and submitting large numbers of mss) are simply more active researchers in all respects, and that these individuals receive relatively few requests to review so can accept and complete a high proportion. Under-contributors may contribute to the peer review system in other ways (e.g., editing for journals). Or, they may allocate their time to other tasks, in particular tasks that they have strong incentives or obligations to perform (e.g., publishing papers). Under-contribution is understandable given the incentives that many researchers face. Academics must “publish or perish”—not “review or perish”.

### Caveats

Our results are from only a small fraction of the peer review system (four journals during 2003–2010). Individuals deviating from balanced interaction with the peer review systems of these journals and time period (i.e., the 1∶1 line in figure 1) would not necessarily deviate so much if all their submitting and reviewing activities were taken into account. However, it is unclear why subsampling of journals would bias our estimates of the fractions of authors over- or under-contributing to the peer review system. Our database lacks reviews performed for other journals by under-contributors, and lacks submissions to other journals by over-contributors—but it also lacks submissions to other journals by under-contributors, and reviews performed for other journals by over-contributors. And we see no reason to think that the individuals submitting to and reviewing for these four journals are unrepresentative of ecologists as a whole in a way that would strongly bias our results. BES journals are among the leading journals in ecology, but we see no reason to think that individuals submitting to and reviewing for leading journals are especially likely to either under- or over-contribute. Hence we expect the patterns presented here to be broadly representative of the wider peer review system in ecology. Future analyses would benefit from: matching of reviews required by a submission to the contribution of the authors to the submitted article, identities (anonymised) of coauthors, and data from as many other journals as possible.

### Future directions and conclusions

Our results may stimulate individuals to consider their own contributions to the peer review system, and their “withdrawals” from it. It is relatively straightforward for individuals to track the required information (at least if one assumes coauthors are doing their share of reviewing), and attempt a balance. Of course, just as individuals may have little incentive to review, they have little incentive to track their own reviewing. But self-tracking requires sufficiently little effort that many might do it without incentives. Furthermore, hiring committees and promotion panels that value evidence of “good citizenship” may like to consider an individual's balance [17]. Adjusting popular metrics of performance, such as the h-index [18], by an individual researcher's peer review balance is possible [12], though necessitates difficult and ultimately arbitrary decisions about the relative weighting of contributing information, and may lead to less clarity than examining multiple metrics individually. If enough researchers were to keep such records, perhaps encouraged by web based tools for doing so, and if they would contribute them to a central database, we could have a better view of the peer review system, and perhaps even pave the way for better recognition of individual's peer review activities.
